# Identification of estrogen-regulated genes by microarray analysis of the uterus of immature rats exposed to endocrine disrupting chemicals

**DOI:** 10.1186/1477-7827-4-49

**Published:** 2006-09-29

**Authors:** Eui-Ju Hong, Se-Hyung Park, Kyung-Chul Choi, Peter CK Leung, Eui-Bae Jeung

**Affiliations:** 1 Laboratory of Veterinary Biochemistry and Molecular Biology, College of Veterinary Medicine, Chungbuk National University, Cheongju, Chungbuk, 361-763, Republic of Korea; 2 Department of Obstetrics and Gynecology, Child and Family Research Institute, University of British Columbia, Vancouver, BC, V6H 3V5, Canada

## Abstract

Environmental estrogenic compounds which bind to the estrogen receptor (ER) can block or alter endogenous functions of estrogen in reproductive and developmental stages. A microarray technology is a very valuable method for the prediction of hormone-responsive activities in various gene expressions. Thus, we investigated the altered gene expression by estrogen and endocrine disruptors (EDs) using microarray technology in the uterus of immature rats. In this study, the expression levels of only 555 genes (7.42%) among the 7636 genes spotted on microarray chips were enhanced by more than two-fold following treatment with estradiol (E2), suggesting that direct or rapid response to E2 is widespread at the mRNA levels in these genes. In addition, elevated expression levels of the genes (over 2-fold) were observed by diethylstilbestrol (DES; 9.01%), octyl-phenol (OP; 8.81%), nonyl-phenol (NP; 9.51%), bisphenol-A (BPA; 8.26%) or genistein (9.97%) in the uterus of immature rats. The expression levels of representative genes, i.e., calbindin-D9k (CaBP-9k; vitamin D-dependent calcium-binding protein), oxytocin, adipocyte complement related protein (MW 30 kDa), lactate dehydrogenase A and calcium binding protein A6 (S100a6; calcyclin), were confirmed in these tissues by real-time PCR. In addition, the mRNA levels of these genes by real-time PCR were increased at follicular phase when E2 level was elevated during estrous cycle of adult female rats. In conclusion, these results indicate distinct altered expression of responsive genes following exposure to E2 and estrogenic compounds, and implicate distinct effects of endogenous E2 and environmental endocrine disrupting chemicals in the uterus of immature rats.

## Background

Environmental chemicals that disrupt endocrine function are suspected for their adverse effects on the reproductive system in wild animals and humans and are being increasingly assumed for their possible participation in inducing estrogenic effects. Furthermore, they are proposed to possess hormone-like properties, i.e., mimicking natural hormones, inhibiting the action of hormones, and inducing abnormal gene expressions. Environmental estrogenic compounds that bind to the estrogen receptors (ERs) can block or alter endogenous estrogen functions in reproductive and developmental stages via an ER-mediated response [1]. Examples of suspected environmental estrogenic chemicals (endocrine disruptors; EDs) include polychlorinated hydroxybiphenyls, DDT and its derivatives, certain insecticides and herbicides (kepone and methoxychlor), plastic components (bisphenol A) and some components of detergents and their biodegradation products (alkylphenols etc.). Although the activity of most of these environmental estrogens is low when compared to endogenous or synthetic estrogens (17β-estradiol; E2 or ethinylestradiol), dietary or environmental exposure scenarios that led to the detection of significant quantities of these substances in human urine and tissue sample have been described [2].

The profound effects of E2 on cell growth, differentiation, and general homeostasis of reproductive and other systems are mediated mainly by the temporal and cell type-specific expression of different genes, whose products are the molecules controlling these molecular events [3,4]. In rats, the concentration of E2 is consistently low throughout neonatal development and starts to increase after day 28 of age [5]. The uterus and ovaries are two of the most sensitive tissues to E2, and both tissues express two forms of ER, ERα and ERβ. In particular, ERα is predominantly expressed in uteri while ERβ is expressed in ovaries [6,7]. Diethylstilbestrol (DES) is a synthetic estrogen which can induce various reproductive alterations in humans [8,9] and mice [10-12]. Numerous reproductive changes in wildlife populations can be caused by EDs which resemble DES [2]. Alkylphenols (APs), such as octyl-phenol (OP) and nonylphenol (NP), were reported to bind directly to the ER in trout, stimulate citelogenin gene expression in trout hepatocytes, be mitogenic in MCF-7 cells and stimulate transcription in mammalian cells via ER [12]. In vitro studies revealed that OP and NP are the most potent estrogenic alkylphenols, and the potency of OP has been shown to be approximately 10^-3^~10^-7 ^relative to 17β-estradiol [12-15]. Furthermore, the binding affinity of BPA indicated that it is approximately 10000-fold less potent than E2 and 20000-fold less potent than DES for both ERα and ERβ receptors [16]. *In vivo *estrogenic activities (400–1000 mg/kg/day) in immature or ovariectomized rats and mice have been recognized. Thus, the concentrations of EDs such as OP, NP, and BPA, in the present study are expected to have similar effects as those of steroid hormones. In addition, APs are weakly estrogenic in traditional uterotropic assays as evidenced by the increase in uterus weight [17]. In vitro assays, E2 has been demonstrated to induce maximal proliferation of MCF-7 cells at 1 nM concentration, and OP and NP have been found to be considerably potent compounds as estrogenic chemicals at 1 and 10 μM, respectively. Treatments with OP and NP inhibited the binding affinity of E2 to ER in MCF-7 cells by a competitive ER binding assay [18]. Bisphenol A (BPA) is a particularly important environmental estrogen. Not only in it widespread in the environment, but it is commonly ingested by humans, being released by polycarbonate plastics, the lining of food cans, and dental sealants [19]. BPA only acts as an agonist of estrogen via ERβ whereas it has dual actions as an agonist and antagonist in some types of cells via ERα Thus, the activity of BPA may depend on the ER subtype and the tissue involved [20]. ERβ has a higher relative binding affinity to genistein (Gen) in *in vitro *assays compare with ERα [6]. Genistein is readily absorbed [21,22] and act as a pharmacological estrogen both *in vitro *and *in vivo *via ERs [23-25]. Although an actual impact of environmental estrogens on reproductive health is not well defined thoroughly, these chemicals have the potential to disrupt the reproductive system and confirm its estrogen-like activity *in vitro *[6]. Therefore, the changes in the expression of estrogen target genes are considered to be a useful index for evaluating the estrogenic activity of synthetic compounds. However, it is difficult to predict the full range of effects of estrogenic compounds from changes in the expression levels of only well-known estrogen target genes.

Reproductive organs are highly susceptible to hormonal exposure during organ development and sex differentiation. In our previous studies, we showed that NP, OP and BPA have estrogenic activity, resulting in uterotrophic effects in the uterus of rats treated with these compounds [26,27]. In addition, we demonstrated that the CaBP-9k gene is not expressed in the uterus of immature rats, which do not obtain estrogen from ovaries, and that it is regulated through the binding of the ER/estrogen complex to the estrogen response element (ERE) in rats [26,28]. CaBP-9k mRNA in the uterus is known to fluctuate during estrous cycle of rats depending on serum estrogen level. CaBP-9k mRNA at diestrus was not detectable, but increased at proestrus and reached the highest level at estrus and then decreased as metestrus [29]. The aim of this study was to identify estrogen-responsive genes by E2 and endocrine disrupting chemicals in the uterus of rats and to determine whether estrogen responsive genes are differentially regulated following exposure to these estrogenic compounds by microarray analysis and real-time PCR. Finally, we evaluated the correlations between E2-induced and EDs-induced gene profiles, and confirmed the biomarker among altered gene expression for screening potential EDs.

## Materials and methods

### Animals and treatments

Immature Sprague-Dawley rats (2-weeks of age) with dams were obtained from Orient Co, Ltd. (Gyeonggi-do, Korea). All animals were housed in polycarbonate cages, and used after acclimation to an environmentally controlled room (temperature: 23 ± 2°C, relative humidity: 50 ± 10%, frequent ventilation and a 12 h day/night cycle). To determine the effect of EDs, each group of five animals (14-days old) was injected subcutaneously (*sc*, 0.1 ml per rat) with E2 (40 μg/kg BW; Sigma-Aldrich Corp, St. Louis, MO, USA), DES (500 μg/kg BW; Sigma-Aldrich Corp), OP (600 mg/kg BW; Fluka Chemie, Buchs, Switzerland), NP (600 mg/kg BW; Sigma-Aldrich Corp), BPA (600 mg/kg BW; Sigma-Aldrich Corp), and genistein (40 mg/kg BW; Sigma-Aldrich Corp) by a single dose daily for 3 days and euthanized 24 h after final injection. All chemicals were dissolved in corn oil (Sigma-Aldrich Corp) as a vehicle. The rats were injected with E2 (*n *= 3) as a positive control or corn oil (*n *= 3) as a negative control daily for 3 days. The uteri were washed in cold sterile 0.9% NaCl solution (0.9% normal saline) and used for microarray and RT-PCR analyses. All rats were euthanized at 24 h after the injection.

It is believed that exposure to DES or endocrine disruptors might be detrimental to development and differentiation, although the effects may not be apparent until adulthood. In the present study, treatment with estrogenic compounds induced a significant increase in the mRNA expression of specific genes in the neonate rat uterus. To confirm altered gene expression profile by E2 or EDs in the uterus of immature rats, adult female rats were also employed to examine the elevated endogenous E2 in the induction of these genes at proestrus and estrus during estrous cycle. Estrous cycle was determined by the observation of three types of the cells derived from the vaginal smears: leukocytes, cornified cells and nucleated epithelial cells. Four stages of the estrous cycle (proestrus, estrus, metestrus and diestrus) were determined as following criteria: proestrus was characterized by many epithelial cells and few leukocytes; estrus by many cornified cells and no leukocytes; metestrus by some cornified cells and many leukocytes; and diestrus by few epithelial cells and many leukocytes. All animals were smeared daily, and the rats that had three regular cycles were selected. Forty female rats were randomly assigned to four groups according to the respective phase of estrous cycle (proestrus, estrus, metestrus and diestrus, n = 10 each) and euthanized immediately. The uteri were washed in cold sterile 0.9% NaCl solution (0.9% normal saline) and used for RNA extraction. All experimental procedures and animal use were approved by the Ethics Committee of the Chungbuk National University.

### RNA isolation and cDNA microarray analysis

Total RNA was extracted with Trizol (Invitrogen, Carlsbad, CA, USA) according to manufacturer's suggested procedure, and purified using RNeasy total RNA isolation kit (Qiagen, Valencia, CA, USA) according to the manufacturer's instructions. DNA was digested using an RNase-free DNase set (Qiagen) during RNA purification. Total RNA was quantified by spectrophotometer and its integrity was assessed by running on a 0.8% agarose gel. To make cDNAs from mRNAs for microarray analysis, the same quantity of each RNA sample from the treated groups (n = 5) or control groups (n = 3) was pooled. A cDNA microarray consists of 7636 cDNA spot including Incyte clones, housekeeping genes and Arabidopsis DNA as controls. The experiments were performed on the rat cDNA microarray prepared as previously described [30]. The PCR reactions were prepared according to the standard protocol and reaction mixtures were subjected to be amplified at 35 cycles. The primer pair used for amplification was overlap primer-1 (5'-AAT TAA CCC TCA CTA AAG GG-3') and overlap primer-2 (5'-GTA ATA CGA CTC ACT ATA GGG C-3'). The size and amount of the PCR products were verified on 1% agarose gel. The PCR products were purified by ethanol precipitation, then resuspended in 15 μl of hybridization solution (GenoCheck, Korea), and spotted onto CMT-GAPS α silane slide glass (Corning, NY) with a pixsys 5500 arrayer (Cartesian Technologies, CA) using 16-Stealth Micro spotting pins. The printed slides were processed according to CMT-GAPS α slide protocol. Briefly, the spots were re-hydrated with 1 × SSC for 1 min and then DNA linked using a UV crosslinker (Stratagene, CA). The slides were soaked in the succinic anhydride/sodium borate solution for 15 min with gentle agitation and then transferred to a 95°C water bath for 2 min. The slides were quickly transferred to 95% ethanol for 1 min and then dried using a centrifuge at 3000 rpm for 20 sec.

### Hybridization with fluorescent DNA probe

Total RNA was extracted from the treated and untreated tissues from immature or adult rats at the indicated time points using the TRI-REAGENT (MRC, OH) according to the manufacturer's instructions. Fluorescent labeled cDNA probes were prepared from 50 §P of total RNA by oligo (dT)_18_-primed polymerization using SuperScript α reverse transcriptase (Invitrogen, NY) in a total reaction volume of 30 μl. The reverse transcription mixture included 400 U Superscript RNase H-reverse transcriptase (Invitrogen), 0.5 mM dATP, dTTP and dGTP, 0.2 mM dCTP and 0.1 mM Cy3 or Cy5 labeled dCTP (NEN Life Science Product Inc.). After reverse transcription, the sample RNA was degraded by adding 5 μl of stop solution (0.5 M NaOH/50 m EDTA) and incubating at 65°C for 10 min. The labeled cDNA mixture was then concentrated using the ethanol precipitation method. After determining the target cDNA quality, cDNA samples derived from the pooled uteri of five individual neonate rats from each treated group were selected and hybridized. The concentrated Cy3 and Cy5 labeled cDNAs were resuspended in 10 μl of hybridization solution (GenoCheck). After two labeled cDNAs were mixed, the mixture was denaturized 95°C for 2 min and then incubated in 45°C water chamber for 20 min. The cDNA mixture was then placed at three spotted slide positions and covered by a cover slip to assess the overall quality of each sample. The slides were hybridized for 12 h at 62°C hybridization chamber. The hybridized slides were washed in 2 × SSC, 0.1% SDS for 2 min, 1 × SSC for 3 min, and then 0.2 × SSC for 2 min at room temperature. The slides were centrifuged at 3000 rpm for 20 sec to be dried.

### Scanning and image analysis

Hybridized slides were scanned with the Axon Instruments GenePix 4000B scanner and the scanned images were analyzed with the software program GenePix Pro 5.1 (Axon, CA) and GeneSpring 7.1 (Sillicongenetics, CA). In order to allow algorithm to eliminate all bad spots, no data points were eliminated by visual inspection from the initial GenePix image. For signal normalization, housekeeping genes (β-actin) and positive control genes (A. thaliana genes) were spotted onto each slide. The signals of these spots were used for normalization. To filter out the unreliable data, spots with signal-to-noise (signal – background – background SD) below 100 were not included in the data. Data were normalized by global, lowess, print-tip and scaled normalization for data reliability. Data were sorted of above 2-fold altered genes using GeneSpring 7.1 (Sillicongenetics) and a hierarchical clustering analysis was performed using Pearson correlation. The statistical significance of differential expression was assessed by computing a q-value for each gene. To determine the q-value, we used a permutation procedure, and for each permutation a two-sample *t*-statistic was computed for each gene. The result was considered significant when the logarithmic gene expression ratio of three independent hybridizations was more than twofold the difference in the expression level. The accuracy of microarray analysis in this study was confirmed by real-time PCR.

### Confirmation of microarray analysis with real-time PCR

The standard curve was generated for a standard RNA preparation by serial dilution (1, 1/10, 1/100, 1/1000, 0). A real-time PCR reaction was carried out in a 25 μl final volume containing 12.5 μl of 2× premix (TaKaRa Bio Inc.), 0.3 μl of each of forward and reverse primers, 1 μl of cDNA, and distilled water up to 10.9 μl. The oligonucleotide sequences of primers were employed to detect various genes as shown Table 1. Polymerase chain reaction of amplification using the Smart Cycle System (TaKaRa Bio Inc.) began with an initial denaturation at 95°C for 30 sec. Each of the 35 amplification cycles consisted of denaturation at 95°C for 5 sec, annealing at 55°C for 15 sec, and extension at 72°C for 15 sec. Relative expression levels of each sample were calculated based on the cycle threshold (Ct) and monitored for an amplification curve. The PCR amplification curves were evaluated by fluorescence of the double-stranded DNA-specific dye, SYBR Green, versus the amount of standardized PCR product. All gene expressions were normalized to Cytochrome oxidase subunits I mRNA (*IA*, Housekeeping gene) as controls.

**Table 1:** Oligonucleotide sequences with predicted sizes of respective PCR product

**Accession No.**	**Gene name**	**Forward sequence**	**Reverse sequence**	**Size**
NM_012521	vitamin D-dependent calcium binding protein	**5'-aagagcatttttcaaaaata-3'**	**5'-gtctcagaatttgctttatt-3'**	314
NM_012996	Oxcytocin	**5'-gaccttcatcatcgtactgg-3'**	**5'-gagttgctcttcttgctgac-3'**	275
NM_144744	Adipocyte complement related protein of 30 kDa	**5'-gtgttcttggtcctaagggt-3'**	**5'-tgtacaccgtgatgtggtag-3'**	287
NM_017025	Lactate dehydrogenase A	**5'-aggtgacactgactcctgac-3'**	**5'-gtgggattgtcacactaacc-3'**	285
AF140232	S100 calcium binding protein A6 (calcyclin)	**5'-cttctcgtggctatcttcc-3'**	**5'-actggacttgactgggatag-3'**	289

### Data analysis

Data are presented as the mean ± SD. The data were analyzed by non-parametric procedure of the Kruskal Wallis test, followed by Dunnett's test for two-pair comparisons. The each value of Dunnett's test was converted to rank for statistical analysis. All statistical analyses were performed with SAS. *P *< 0.05 was considered statistically significant.

## Results

### Global analysis of estrogen-regulated genes compared with endocrine disruptors

**Figure 1:**
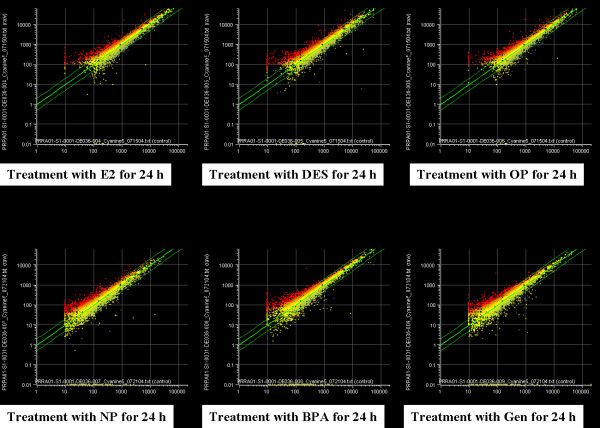
A scattergram of gene expression analyzed by DNA microarray. For each gene, the relative mRNA level in the control is given on the *x *axis and the expression level for the same transcript in the experimental sample (estrogenic compound exposed) is plotted on the *y *axis. Each graph displays two lines indicating 2-fold up-regulation or down-regulation in the expression level of each individual probe set comparing treated *vs*. control sample. The linear line (*P *< 0.05) was fitted to the microarray data.

**Figure 2:**
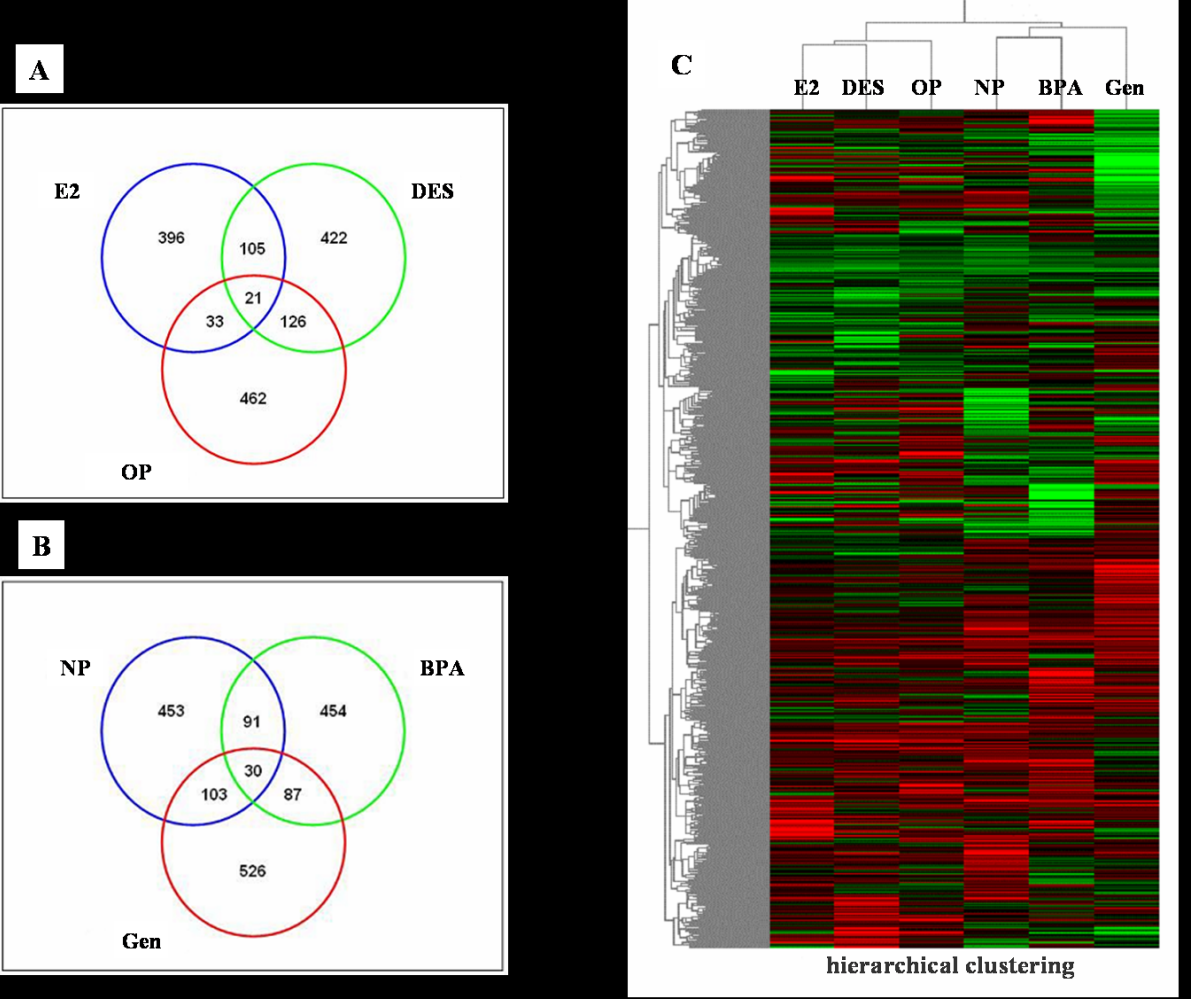
Venn diagram showing the number of genes induced by E2 and EDs. The altered gene profiles by E2, OP and DES (overlapped 21 genes; **A**) or NP, BPA and Gen (overlapped 30 genes **B**), respectively, were summarized. A hierarchical clustering analysis (**C**) was performed following treatments with E2 and other EDs in the uterus of immature rats. Two-dimensional hierarchical clustering was applied to the expression data from 7.5 k genes, which showed significant changes in the balanced differential expression. Increased expression levels are shown in red and decreased expression levels are shown in green.

Following individual treatment of immature rats with EDs, total RNAs from the uteri were isolated and processed by microarray analysis as described. Based on the number of genes expressed in control versus treated samples, the overall pattern of altered gene expression was similar between the control (vehicle-treated) and estrogenic compound-treated (E2, DES, OP, NP, BPA or genistein) rats. Although the number of genes whose expression is altered by any chemical tested is modest, there is substantial change in the gene expression altered or modified by exposure to each compound. The mean values of the intensities of each spot in this study were calculated, and are plotted in Fig. 1. The data shown in Fig. 1 are typical of the three self *vs*. self comparisons, which showed that a more than 2-fold change would be necessary to overcome the intrinsic variability introduced by the hybridization procedure. For this reason, the data was further filtered to select for genes that showed an absolute differential of at least two-fold or greater. Of the 7479 genes examined, the change in the mRNA expressions of 85 genes was listed in the Additional file 1. In addition, treatment with E2 resulted in the induction of 555 genes by more than two-fold. About 7.42% of all genes were up-regulated by E2 (Fig. 2A), causing no significant difference in the expression of the remaining 92.58% of the genes. As shown in Fig. 2A, the expression level significantly increased in the uterus when treated with DES (9.01%) or OP (8.81%). Furthermore, Table 2 shows the common 21 genes induced by E2, DES, and OP in the uterus of immature rats. In addition, a single treatment of NP (9.51%), BPA (8.26%) or genistein (9.97%) induced a significant increase in gene expressions in this tissue (Fig. 2B), and there are 30 common genes by these EDs (Table 3). These results indicate that significant gene profile is altered in the uteri by E2 and EDs.

**Table 2:** Altered genes induced by E2, DES and OP in the uterus of immature rats

**Accession No.**	**Gene name**	**Gene symbol**	**E2**	**DES**	**OP**
AF154245			3.20	4.47	2.66
AF494463	Seizure related 6 homolog (mouse)	Sez6	3.32	2.47	3.44
NM_012843	Epithelial membrane protein 1	Emp1	2.24	2.24	3.21
AW915015	Discs, large homolog 3 (Drosophila)	Dlgh3	2.19	3.29	2.75
AF039584	Decay accelarating factor 1	Daf1	6.35	6.46	8.47
NM_031029	Gamma-aminobutyric acid (GABA) A receptor, pi	Gabrp	2.59	2.90	4.10
NM_133296	X transporter protein 3	Xtrp3	2.25	2.11	4.12
AF020045	Integrin, alpha E, epithelial-associated	Itgae	6.54	3.01	2.15
NM_031698	Ribophorin II	Rpn2	3.45	2.09	3.16
NM_031977	Rous sarcoma oncogenes	Src	2.28	2.29	2.13
NM_021702	Ataxin 3	Atxn3	2.61	5.68	2.79
AF151982	Secretory leukocyte peptidase inhibitor	Slpi	2.11	2.89	2.26
NM_019188	Beta-microseminoprotein	Msmb	2.29	3.14	3.42
NM_019369	Inter alpha-trypsin inhibitor, heavy chain 4	Itih4	2.43	2.47	3.12
U06752	Mucin 4	Muc4	3.99	5.84	4.10
NM_019278	Regulated endocrine-specific protein 18	Resp18	2.37	4.15	3.78
NM_017025	Lactate dehydrogenase A	Ldha	2.17	2.06	2.10
NM_134377	Calsyntenin 2	Cstn2	2.60	2.46	2.42
AJ223184	Immunoglobulin superfamily, member 6	Igsf6	7.61	2.23	2.38
AI556074	Myristoylated alanine rich protein kinase C substrate	Marcks	2.08	2.80	2.43
NM_145788	TRAF family member-associated Nf-kappa B activator	Tank	3.00	3.05	2.82

**Table 3:** Altered genes induced by NP, BPA, and Gen in the uterus of immature rats

**Accession No.**	**Gene name**	**Gene symbol**	**NP**	**BPA**	**Gen**
NM_017224	Solute carrier family 22 (organic anion transporter), member 6	Slc22a6	2.95	2.09	2.12
NM_012500	N-acylaminoacyl-peptide hydrolase	Apeh	3.93	2.53	2.08
U12571	Rabphilin 3A homolog (mouse)	Rph3a	2.48	2.04	4.11
L34821	Aldehyde dehydrogenase family 5, subfamily A1	Aldh5a1	5.27	2.33	2.77
AF053317	Solute carrier family 21, member 1	Slc21a1	2.05	2.21	3.82
AF115282	Inhibitor of kappaB kinase beta	Ikbkb	2.19	2.58	3.64
NM_024484	Aminolevulinic acid synthase 1	Alas1	15.07	3.65	5.16
NM_017208	Lipopolysaccharide binding protein	Lbp	2.68	12.17	4.15
NM_133392	Serine/threonine kinase 17b (apoptosis-inducing)	Stk17b	3.12	3.91	2.18
X55969	Apolipoprotein B	Apob	2.31	2.41	2.35
NM_019333	6-phosphofructo-2-kinase/fructose-2,6-biphosphatase 4	Pfkfb4	2.48	2.81	2.51
NM_031698	Ribophorin II	Rpn2	2.81	3.15	10.86
AJ293948	Kelch repeat and BTB (POZ) domain containing 10	Kbtbd10	2.50	2.43	2.71
D13127	ATP synthase, H+ transporting, O subunit	Atp5o	2.55	3.02	2.40
NM_021689	Epiregulin	Ereg	2.31	3.07	2.32
NM_022709	SMR2	Smr2	8.56	24.93	68.75
BF549833	Transcribed locus, strongly similar to XP_130951.1 dolichyl-phosphate mannosyltransferase polypeptide 3 [Mus musculus]		3.36	2.20	4.24
X59290	Eph and elk-related kinase	LOC60589	2.16	2.75	4.81
BF521799	Transcribed locus, moderately similar to XP_525535.1 similar to Yippee-like protein 1 (DiGeorge syndrome-related protein FKSG3)		5.14	2.72	3.61
NM_031577	Growth hormone releasing hormone	Ghrh	3.72	5.51	2.39
NM_017145	Mast cell protease 1	Mcpt1	2.57	2.19	2.98
U57062	Granzyme C	Gzmc	6.65	2.50	2.08
NM_130421	Lymphocyte cytosolic protein 2	Lcp2	2.72	2.50	2.14
NM_031971	Heat shock 70 kD protein 1A	Hspa1a	3.62	2.25	2.29
M83680	RAB14, member RAS oncogene family	Rab14	3.03	4.36	3.06
AJ132846	Sodium-dependent neutral amino acid transporter ASCT2	Slc1a5	2.77	2.19	2.73
NM_138532	Non-metastatic cells 7, protein expressed in	Nme7	3.63	2.38	2.96
AF168795	CDK107	Slfn3	3.03	3.04	6.29
U22520	Chemokine (C-X-C motif) ligand 10	Cxcl10	2.05	2.19	3.87
NM_031785	ATPase, H+ transporting, lysosomal (vacuolar proton pump), subunit 1	Atp6ap1	3.81	3.52	3.93

In this study, we extended a large-scale of microarray analysis to six other estrogenic compounds, and identified crucial estrogen-responsive genes, which were either common in all or specific to each or some of the compounds. The results with all six compounds are shown in Fig. 2C using a hierarchical clustering algorithm of the mean values of **triplicate **slides and a pseudo-color visualization matrix. The gene profile in red color shows a higher expression than the control, while the gene profile in green color shows a lower expression (Fig. 2C). These genes might be universal estrogen – responsive genes among various tissues. These compounds exhibited a reasonable hierarchical clustering; DES or OP resembled to E2. However, NP-induced gene profile is similar to that of BPA or genistein.

### Confirmation of estrogen-regulated genes by real-time PCR

**Figure 3:**
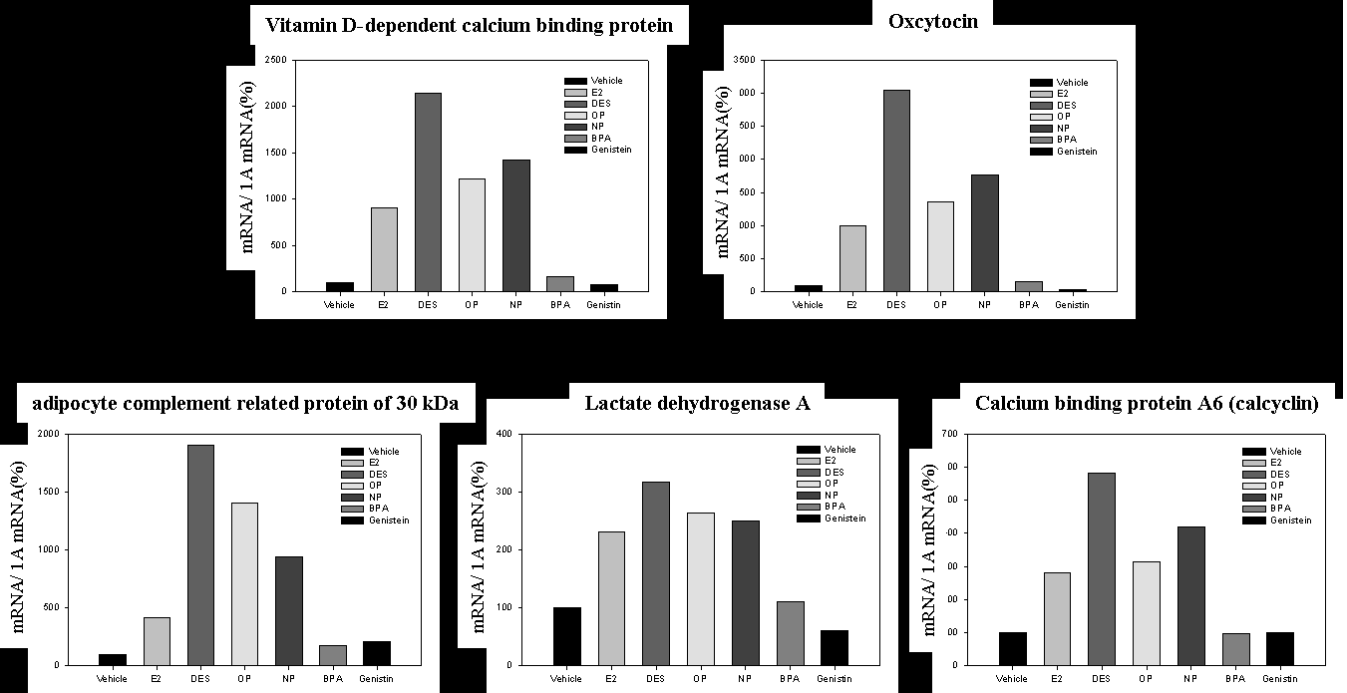
Confirmation of gene profiles by real-time PCR analysis. Relative values of expression of the altered genes quantified by real-time PCR are shown in graphs, indicating the comparison of fold change determined by real-time PCR analysis by E2, DES, OP, NP, BPA, and Gen in the uteri of immature rats. The representative genes are CaBP-9k, oxytocin, adipocyte complement related protein (MW 30 kDa), lactate dehydrogenase A and calcium binding protein A6 (calcyclin). Total RNAs from the uterus following treatment with estrogenic compounds were used to quantify altered gene expression normalized by cytochrome oxidase subunits I (1A) as a control.

The primer sets used in the RT-PCR are listed in Table 1, and the altered genes which showed a high increase in the uterus are listed in Fig. 3. The expression patterns of several genes up-regulated by estrogen were confirmed to verify the results of the microarray analysis using real-time PCR analysis. The rats were injected with endocrine disruptors to determine their effect on the induction of calbindin D9k (CaBP-9k) mRNA in the immature uterus. The expression level of CaBP-9k mRNA significantly increased when treated with OP (12.2-fold vs. vehicle), NP (14.2-fold) and BPA (1.6-fold) for 3 days (Fig. 3). In addition, CaBP-9k mRNA was markedly induced by a single dose of E2 (9.1-fold) or DES (21.4-fold) as seen in Fig. 3. The induction of uterine oxytocin by estrogenic compounds was further assessed by real-time RT-PCR. An increase in oxytocin was observed in the uterus of rats as expected when treated with a single of OP (13.6-fold), NP (17.4-fold) and BPA (1.5-fold) for 3 days Furthermore, treatment with a single dose of E2 (9.9-fold) or DES (30.5-fold) resulted in an increase of oxytocin in this tissue. Increased expression level of adipocyte complement related protein (30 KDa; Acrp30) was observed when treated with OP (14.1-fold), NP (9.4-fold) and BPA (1.7-fold). In addition, a single dose of E2 (4.1-fold) or DES (19.1-fold) induced a marked increase in Acrp30 (Fig. 3). The treatment with E2 (2.3-fold), DES (3.1-fold), OP (2.6-fold), and NP (2.5-fold) altered the expression level of lactate dehydrogenase A (Ldha). As shown in Fig. 3, the expression level of calcium binding protein A6 (S100a6) in this tissue significantly increased when treated with OP (3.1-fold) and NP (4.2-fold). In addition, a single dose of E2 (2.8-fold) and DES (5.8-fold) treatment induced a significant increase in the expression of calcium binding protein A6 mRNA in the uterus of rats. These results indicate that estrogenic compounds in parallel with E2 in the uterus regulate the expression levels of various genes.

### Expression of estrogen-regulated genes during estrous cycle

**Figure 4:**
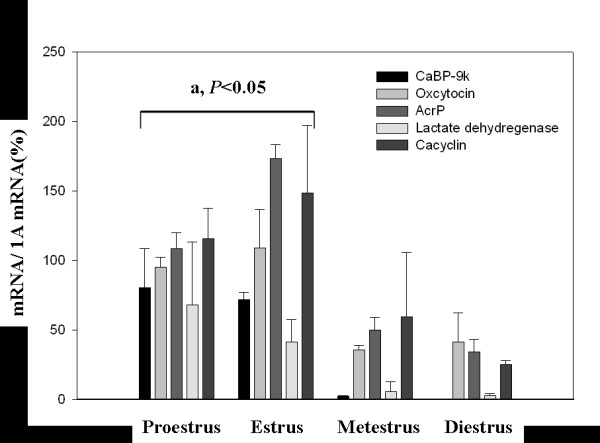
Expression of *CaBP-9k*, oxytocin, Acrp30, Ldha and calcyclin mRNAs in the uterus of adult rats during estrous cycle. To investigate the expression of these genes during estrous cycle, the expression levels of these genes in the uterus of adult female rats were analyzed by real-time PCR. Data were analyzed by non-parametric procedure of the Kruskal Wallis test, followed by Dunnett's test for two-pair comparisons. The values represent means ± SD. a, *P *< 0.05 *vs*. Metestrus and Diestrus.

To investigate the regulation of various genes in the uterus of rats during estrous cycle, the expression levels of CaBP-9k, oxytocin, Acrp30, Ldha, and S100a6 mRNA were analyzed by real-time PCR. They were highly expressed in the uterus in a follicular phase (proestrus and estrus) compared to a luteal phase (metestrus and diestrus) as shown in Fig. 4. The level of CaBP-9k mRNA at follicular phase increased up to 70.5-fold compared to that at luteal phase (Fig. 4). In parallel with CaBP-9k mRNA, the expression levels of oxytocin (13.4-fold, *vs*. luteal phase), Acrp30 (3.1-fold, *vs*. luteal phase), Ldha (3.3-fold, *vs*. luteal phase), and S100a6 (2.7-fold, *vs*. luteal phase) mRNAs at follicular phase were up-regulated in this tissue, indicating that they are dominantly expressed during follicular phase and weakly detected at luteal phase in the uterus of mature rats.

## Discussion

The microarray technique is a very valuable method for the prediction of hormone-responsive activity in various gene expressions. Estrogen plays an important role in various molecular events, but the molecular mechanisms that are regulated by estrogen in the uterus remain largely unknown. Therefore, the identification of estrogen-induced gene expression is essential to understanding how estrogenic compounds regulate uterine physiology and pathology at the cellular level. In the present study, microarray analysis showed that only 7.42% (555 genes) of all genes spotted on the microarray slide were up-regulated by more than two-fold following estrogen treatment, suggesting that direct or rapid responses to estrogen is widespread at the mRNA level. Furthermore, treatments with DES (9.01%), OP (8.81%), NP (9.51%) BPA (8.26%) or genistein (9.97%) showed an induction of distinct genes by more than two-fold in the uterus. Recently, gene profile patterns by microarray technology were determined in the developing uterus and ovaries of Sprague-Dawley rats at different stages of the development exposed to graded dosages (sc) of 17alpha-ethynyl estradiol (EE), Gen, or BPA [31,32]. This analysis of the transcript profile in a dose-dependent manner revealed that a common set of genes are altered, but some of the genes are differentially changed by these estrogenic chemicals [31]. Interestingly, 592 genes of immature rat showed strong and consistent changes in expression after EE exposure [33], indicating that previously identified estrogen-sensitive genes are sensitive to EE exposure and that they are targets of the estrogenic action of EDs in both the uterus and the ovary. In actual, the level of EDs in nature is low compared with using dosage in the present study. For example, several studies have been performed to assess the presence of EDs in milk. EDs were determined in milk and infant formula at concentrations from 0.4 to 81 μg/kg in NP [34], or 0.1 to 13.2 μg/kg in BPA [35], respectively. Although we are aware that we used the high dose of EDs, this study focused to describe estrogen specific genes using cDNA microarray to detect estrogenicity in vivo following treatment with EDs.

Uterine CaBP-9k may be involved in controlling myometrial activity which is affected by the intracellular calcium level; however, the exact role of CaBP-9k in the uterus is still under investigation by us and other research groups [36]. Recently, it has been demonstrated in our previous studies that both CaBP-9k mRNA and protein could be a novel biomarker for estrogenic compounds in the uterus of immature rats [26,27]. Based on the previous results, the present study was performed to further investigate EDs-induced specific gene expression in the uterus following treatment with DES, E2 and estrogenic compounds such as OP, NP, BPA and genistein. Thus, the expression levels of CaBP-9k mRNA were analyzed to confirm the estrogenic effect of these compounds in this tissue. E2 is a major factor controlling *CaBP-9k *gene expression in the rat uterus. The *CaBP-9k *gene is not expressed in the uterus of mature ovariectomized and immature rats which do not have circulating E2 from ovaries [37]. Using microarray, the expression level of uterine CaBP-9k mRNA was significantly increased when treated with E2 (4.2-fold), DES (7.32-fold), OP (3.09-fold), and NP (2.57-fold) in the uterus of immature rats [see Additional file 1]. In pregnant rats, a relative potency of estrogenic compounds indicated OP = NP > BPA [38]. Despite different potency in estrogenicity, E2 and the estrogenic compounds tested in this study induced a significant increase of CaBP-9k mRNA in the uterus of immature rats. The expression profile potentially provides a wealth of data about the differences in gene expression between experimental samples; however, these differences do not always reflect realistic mRNA levels. The induction of uterine CaBP-9k by estrogenic compounds was further assessed by real-time RT-PCR. Although the gene expression levels by microarray analysis were not identical to those obtained PCR analysis, the expression patterns of these genes obtained by these two types of analysis were largely similar. In agreement with a previous study [29], there was no change in the expression pattern of CaBP-9k mRNA during the estrous cycle. Estrogen stimulated the number of uterine oxytocin binding sites, and oxytocin receptor mRNA expression in ovariectomized virgin rats [39,40]. In rats, during the terminal stages of pregnancy, the myometrium is extremely sensitive to oxytocin and this increase in uterine responsiveness occurs in parallel with increases in the number of uterine oxytocin binding sites [39]. This leads to increased uterine responsiveness to oxytocin and the onset of the uterine contractions that facilitate parturition. The change in uterine responsiveness to oxytocin involves an increase in the quantity of oxytocin receptor protein per cell and the number of smooth muscle cells that express oxytocin receptors [41]. Based on the treatments with EDs, increased expression levels of oxytocin mRNA were observed when rats were treated with OP (14.76-fold) and NP (9.54-fold), and a single dose treatment with DES (5.70-fold) and E2 (5.83-fold) for 3 days. However, treatments with BPA and genistein for 3 days failed to detect the expression level of oxytocin mRNA [see Additional file 1]. These observations raise the possibility that the increase in the oxytocin mRNA level shown in Figure 3, occurs, at least in part, as a result of the injected estrogenic compounds and that the PCR data and microarray data are in general agreement. Furthermore, the present study indicated at least that oxytocin mRNA which can be amplified with the primer set increase about 2-fold on proestrus compared with the value on metestrus. The mRNA levels of oxytocin increased 2-fold between metestrus and proestrus by Northern blot analysis during estrous cycle, while oxytocin binding increased more than 10-fold within this same interval in the uterus of rats [40].

In the present study, the expression level of adiponectin (adipocyte complement-related protein of 30 kDa, Acrp30) in the uterus of rats increased in microarray analysis following with E2 (5.86-fold), DES (6.26-fold), OP (5.97-fold), and NP (10.05-fold) treatments. Using real-time PCR analysis, we confirmed the expression of Acrp30 mRNA in the neonatal uteri following injection with EDs. Although the rates of Acrp induction were not identical from microarray analysis, treatment with E2, DES, OP and NP resulted in significant increases in Acrp30 mRNA. Furthermore, our results showed that Acrp30 fluctuated during the estrous cycle, suggesting that steroid hormones play a role in the regulation of Acrp30. Acrp30 is expressed exclusively in the adipocytes, its hormone is exclusively secreted by differentiated adipocytes [42], and its protein was secreted and detected in plasma [43]. The levels of the adipocyte hormones, leptin and adiponectin, appear to be correlated with the cell proliferation index and sex steroid receptor abundance [44]. Furthermore, OVX in young cycling mice induced plasma Acrp30, and E2 implants reversed the effect [45]. However, our results indicate that E2, DES, OP and NP up-regulated its level, suggesting that it does not imply any reverse effect in this tissue.

The lactate dehydrogenase-A (Ldha) is hormonally regulated in rodents and in highly expressed in the rat mammary gland during pregnancy and lactation, and Ldha mRNA also increases during mammary gland tumorigenesis [46]. E2 induces lactate dehydrogenase activity in MCF-7 human breast cancer cells, and is elevated in estrogen receptor positive or progesterone receptor positive tumors [47]. The synthesis of Ldha isoenzyme was found to increase significantly in the uterus of immature mice, and expression from the mouse lactate dehydrogenase-A promoter fused to the cat gene in Chinese hamster ovary cells was also induced by E2 and DES [48]. Using microarray, the expression level of uterine Ldha mRNA was significantly increased when treated with E2 (2.17-fold), DES (2.06-fold), OP (1.77-fold), and NP (2.72-fold) in the uterus of immature rats [see Additional file 1]. In addition, we further investigated the expression of Ldha mRNA related to estrogen during estrus cycle. A significant increase in Ldha mRNA was detected at proestrus and estrus compared with metestrus and diestrus. The expression pattern of Ldha mRNA during the estrous cycle was in parallel with CaBP-9k mRNA, whereas others appeared to peak at estrus compared to their level at proestrus.

Calcyclin (S100A6), a small acidic protein that weighs about 10 kDa, belongs to the S100 calcium-binding protein family [49]. These family members share a common S100 calcium-binding motif and are implicated in several regulatory functions that include protein phosphorylation, some enzyme activities, the dynamics of cytoskeletal components, transcription factors, and Ca^2+ ^homeostasis, and also cell proliferation and differentiation [50]. The effect of E2 on the expression level of calcyclin mRNA in uteri was further examined to elucidate a relationship between expression levels of calcyclin and estrogenic compounds. E2 resulted in an induction of uterine calcyclin mRNA in immature rats. In addition, treatment with estrogenic compounds resulted in a significant increase in the expression of calcyclin mRNA, whose level paralleled those of oxytocin and Acrp30, as shown in the Additional file 1 and Fig. 3. After adjusting the data, it is clear that major alterations in gene profiles were induced by estrogenic compounds, such as E2, DES, OP, and NP, and that these differences could have a significant effect on uterine function in reproductive tissues during the estrous cycle.

Taken together, we demonstrated that an alteration in various mRNAs of gene profiles is one of the most significant factors at the transcriptional level in the reproductive tissue following E2, DES or estrogenic compounds. In addition, the expression patterns of CaBP-9k, oxytocin, Acrp, Ldha, and calcyclin mRNAs were altered in the uterus of immature rats during the estrous cycle. In conclusion, these results indicate distinct altered expression of responsive genes following exposure to estrogen and estrogenic compounds, and implicate differential effects of estrogen and environmental endocrine disrupting chemicals in the uterus of immature rats.

## Supplementary Material

Additional file 1 Altered gene expressions induced by E2, DES, OP, NP, BPA and Gen in the uterus of immature rats. Microarray analysis in the uterus of immature rats following treatments with E2, DES, OP, NP, BPA or Gen Click here for file
