# Supplementary material for: Identification of estrogen-regulated genes by microarray analysis of the uterus of immature rats exposed to endocrine disrupting chemicals
Source: Reprod Biol Endocrinol. 2006 Sep 29;4:49. doi: 10.1186/1477-7827-4-49 (PMC1594574; doi:10.1186/1477-7827-4-49)
Supplement: Additional file 1 — Altered gene expressions induced by E2, DES, OP, NP, BPA and Gen in the uterus of immature rats. Microarray analysis in the uterus of immature rats following treatments with E2, DES, OP, NP, BPA or Gen [file 1477-7827-4-49-S1.doc]

**Altered gene expressions induced by E2, DES, OP, NP, BPA and Gen in the uterus of immature rats**

| **Accession**  **No.** | **Gene name** | **Gene symbol** | **E2** | **DES** | **OP** | **NP** | **BPA** | **Gen** |
| --- | --- | --- | --- | --- | --- | --- | --- | --- |
| [D82928](http://www.ncbi.nlm.nih.gov/entrez/viewer.fcgi?val=D82928&view=gb) | CDP-diacylglycerol—inositol 3-phosphatidyltransferase | [Cdipt](http://genecards.curie.fr/cgi-genecards/cardsearch.pl?search=Cdipt) | 11.81 | 0.77 | 1.71 | 2.64 | 1.67 | 2.10 |
| [L34821](http://www.ncbi.nlm.nih.gov/entrez/viewer.fcgi?val=L34821&view=gb) | Aldehyde dehydrogenase family 5, subfamily A1 | [Aldh5a1](http://genecards.curie.fr/cgi-genecards/cardsearch.pl?search=Aldh5a1) | 11.25 | 3.20 | 6.33 | 3.72 | 5.51 | 2.39 |
| [NM_012584](http://www.ncbi.nlm.nih.gov/entrez/viewer.fcgi?val=NM_012584&view=gb) | Steroid delta-isomerase, 3 beta | [Hsd3b](http://genecards.curie.fr/cgi-genecards/cardsearch.pl?search=Hsd3b) | 10.01 | 1.40 | 1.47 | - | - | 0.69 |
| [AF106657](http://www.ncbi.nlm.nih.gov/entrez/viewer.fcgi?val=AF106657&view=gb) | Ubiquitin specific protease 15 | [Usp15](http://genecards.curie.fr/cgi-genecards/cardsearch.pl?search=Usp15) | 9.80 |  | 0.87 | 0.43 | 4.62 | - |
| [U56822](http://www.ncbi.nlm.nih.gov/entrez/viewer.fcgi?val=U56822&view=gb) | Killer cell lectin-like receptor subfamily A, member 22 | [Klra22](http://genecards.curie.fr/cgi-genecards/cardsearch.pl?search=Klra22) | 9.67 | 1.09 | 4.20 | 5.70 | 2.19 | 0.23 |
| [AB079673](http://www.ncbi.nlm.nih.gov/entrez/viewer.fcgi?val=AB079673&view=gb) | Fibroblast growth factor 4 | [Fgf4](http://genecards.curie.fr/cgi-genecards/cardsearch.pl?search=Fgf4) | 8.62 | 3.42 | 1.16 | - | - | 2.61 |
| [BF289012](http://www.ncbi.nlm.nih.gov/entrez/viewer.fcgi?val=BF289012&view=gb) | Hook homolog 3 | [Hook3](http://genecards.curie.fr/cgi-genecards/cardsearch.pl?search=Hook3) | 8.30 | 1.00 | 4.58 | - | 2.32 | 0.99 |
| [NM_031614](http://www.ncbi.nlm.nih.gov/entrez/viewer.fcgi?val=NM_031614&view=gb) | Thioredoxin reductase 1 | [Txnrd1](http://genecards.curie.fr/cgi-genecards/cardsearch.pl?search=Txnrd1) | 7.83 | 12.49 | 15.40 | 8.56 | 24.93 | 68.75 |
| [AF322217](http://www.ncbi.nlm.nih.gov/entrez/viewer.fcgi?val=AF322217&view=gb) | Immunoglobulin superfamily, member 1 | [Igsf1](http://genecards.curie.fr/cgi-genecards/cardsearch.pl?search=Igsf1) | 7.35 | - | 7.17 | 0.55 | 1.05 | - |
| [NM_031338](http://www.ncbi.nlm.nih.gov/entrez/viewer.fcgi?val=NM_031338&view=gb) | Calcium/calmodulin-dependent protein kinase kinase 2, beta | [Camkk2](http://genecards.curie.fr/cgi-genecards/cardsearch.pl?search=Camkk2) | 7.03 | 1.02 | 1.07 | 3.36 | - | 1.40 |
| [NM_012961](http://www.ncbi.nlm.nih.gov/entrez/viewer.fcgi?val=NM_012961&view=gb) | G protein-coupled receptor 1 | [Gpr1](http://genecards.curie.fr/cgi-genecards/cardsearch.pl?search=Gpr1) | 6.89 | 1.54 | 0.63 | - | 1.32 | - |
| [AJ276774](http://www.ncbi.nlm.nih.gov/entrez/viewer.fcgi?val=AJ276774&view=gb) | RAS protein-specific guanine nucleotide-releasing factor 2 | [Rasgrf2](http://genecards.curie.fr/cgi-genecards/cardsearch.pl?search=Rasgrf2) | 6.59 | 2.98 | 1.16 | 0.65 | 4.66 | 0.68 |
| [AF020045](http://www.ncbi.nlm.nih.gov/entrez/viewer.fcgi?val=AF020045&view=gb) | Integrin, alpha E, epithelial-associated | [Itgae](http://genecards.curie.fr/cgi-genecards/cardsearch.pl?search=Itgae) | 6.54 | 3.01 | 1.03 | 0.36 | 3.71 | 2.98 |
| [AF245040](http://www.ncbi.nlm.nih.gov/entrez/viewer.fcgi?val=AF245040&view=gb) | Dickkopf homolog 3 (Xenopus laevis) | [Dkk3](http://genecards.curie.fr/cgi-genecards/cardsearch.pl?search=Dkk3) | 6.37 | 2.70 | 3.81 | 0.79 | 2.27 | 3.09 |
| [U52102](http://www.ncbi.nlm.nih.gov/entrez/viewer.fcgi?val=U52102&view=gb) | Collapsin response mediator protein 1 | [Crmp1](http://genecards.curie.fr/cgi-genecards/cardsearch.pl?search=Crmp1) | 6.26 | 0.88 | 2.57 | 10.77 | 1.49 | 0.42 |
| [U48220](http://www.ncbi.nlm.nih.gov/entrez/viewer.fcgi?val=U48220&view=gb) | Cytochrome P450, family 2, subfamily d, polypeptide 22 | [Cyp2d22](http://genecards.curie.fr/cgi-genecards/cardsearch.pl?search=Cyp2d22) | 6.21 | 1.12 | 1.80 | 7.26 | 0.98 | 2.50 |
| [NM_144744](http://www.ncbi.nlm.nih.gov/entrez/viewer.fcgi?val=NM_144744&view=gb) | Adipocyte complement related protein of 30 kDa | [Acdc](http://genecards.curie.fr/cgi-genecards/cardsearch.pl?search=Acdc) | 5.86 | 6.26 | 5.97 | 10.05 | 1.41 | 1.12 |
| [NM_012996](http://www.ncbi.nlm.nih.gov/entrez/viewer.fcgi?val=NM_012996&view=gb) | Oxcytocin | [Oxt](http://genecards.curie.fr/cgi-genecards/cardsearch.pl?search=Oxt) | 5.83 | 5.70 | 14.76 | 9.54 | - | - |
| [U72353](http://www.ncbi.nlm.nih.gov/entrez/viewer.fcgi?val=U72353&view=gb) | Lamin B1 | [Lmnb1](http://genecards.curie.fr/cgi-genecards/cardsearch.pl?search=Lmnb1) | 5.77 | 1.22 | 2.75 | - | 1.04 | 0.70 |
| [NM_019246](http://www.ncbi.nlm.nih.gov/entrez/viewer.fcgi?val=NM_019246&view=gb) | Proprotein convertase subtilisin/kexin type 7 | [Pcsk7](http://genecards.curie.fr/cgi-genecards/cardsearch.pl?search=Pcsk7) | 5.45 | 0.69 | 1.93 | 2.44 | 1.00 | 3.26 |
| [NM_012891](http://www.ncbi.nlm.nih.gov/entrez/viewer.fcgi?val=NM_012891&view=gb) | Acyl-Coenzyme A dehydrogenase, very long chain | [Acadvl](http://genecards.curie.fr/cgi-genecards/cardsearch.pl?search=Acadvl) | 5.35 | 1.88 | 2.19 | 1.63 | 3.77 | 1.87 |
| [NM_024136](http://www.ncbi.nlm.nih.gov/entrez/viewer.fcgi?val=NM_024136&view=gb) | Epididymal retinoic acid-binding protein | [Erabp](http://genecards.curie.fr/cgi-genecards/cardsearch.pl?search=Erabp) | 5.19 | 1.63 | 1.62 | 1.92 | 2.34 | 1.14 |
| [NM_013022](http://www.ncbi.nlm.nih.gov/entrez/viewer.fcgi?val=NM_013022&view=gb) | Rho-associated coiled-coil forming kinase 2 | [Rock2](http://genecards.curie.fr/cgi-genecards/cardsearch.pl?search=Rock2) | 4.97 | 0.57 | 3.03 | 0.40 | 0.99 | - |
| [AF003835](http://www.ncbi.nlm.nih.gov/entrez/viewer.fcgi?val=AF003835&view=gb) | Isopentenyl-diphosphate delta isomerase | [Idi1](http://genecards.curie.fr/cgi-genecards/cardsearch.pl?search=Idi1) | 4.91 | 2.17 | 2.55 | 3.14 | 1.64 | 0.23 |
| [NM_031579](http://www.ncbi.nlm.nih.gov/entrez/viewer.fcgi?val=NM_031579&view=gb) | Protein tyrosine phosphatase 4a1 | [Ptp4a1](http://genecards.curie.fr/cgi-genecards/cardsearch.pl?search=Ptp4a1) | 4.86 | 3.56 | 1.36 | 3.19 | 2.23 | 1.00 |
| [U97667](http://www.ncbi.nlm.nih.gov/entrez/viewer.fcgi?val=U97667&view=gb) | round spermatid protein RSP29 gene |  | 4.77 | - | 8.47 | 2.19 | 0.94 | - |
| NM_017018 | Histamine receptor H 1 | [Hrh1](http://genecards.curie.fr/cgi-genecards/cardsearch.pl?search=Hrh1) | 4.56 | 3.33 | 0.55 | 1.87 | 5.63 | 0.83 |
| [NM_013014](http://www.ncbi.nlm.nih.gov/entrez/viewer.fcgi?val=NM_013014&view=gb) | Persephin | [Pspn](http://genecards.curie.fr/cgi-genecards/cardsearch.pl?search=Pspn) | 4.50 | 2.66 | 4.10 | - | - | - |
| [L31883](http://www.ncbi.nlm.nih.gov/entrez/viewer.fcgi?val=L31883&view=gb) | Tissue inhibitor of metalloproteinase 1 | [Timp1](http://genecards.curie.fr/cgi-genecards/cardsearch.pl?search=Timp1) | 4.47 | 1.21 | 2.42 | 3.51 | 1.49 | 1.70 |
| [NM_022962](http://www.ncbi.nlm.nih.gov/entrez/viewer.fcgi?val=NM_022962&view=gb) | Latrophilin 1 | [Lphn1](http://genecards.curie.fr/cgi-genecards/cardsearch.pl?search=Lphn1) | 4.37 | 2.71 | 0.90 | 0.66 | 5.26 | 3.03 |
| [NM_012521](http://www.ncbi.nlm.nih.gov/entrez/viewer.fcgi?val=NM_012521&view=gb) | Calbindin 3, (vitamin D-dependent calcium binding protein) | [Calb3](http://genecards.curie.fr/cgi-genecards/cardsearch.pl?search=Calb3) | 4.25 | 7.32 | 3.09 | 2.57 | 0.88 | 0.44 |
| [NM_031728](http://www.ncbi.nlm.nih.gov/entrez/viewer.fcgi?val=NM_031728&view=gb) | Synaptosomal-associated protein, 91kDa homolog (mouse) | [Snap91](http://genecards.curie.fr/cgi-genecards/cardsearch.pl?search=Snap91) | 4.23 | 6.24 | 2.42 | - | 0.73 | 0.77 |
| [AB071986](http://www.ncbi.nlm.nih.gov/entrez/viewer.fcgi?val=AB071986&view=gb) | ELOVL family member 6, elongation of long chain fatty acids | [Elovl6](http://genecards.curie.fr/cgi-genecards/cardsearch.pl?search=Elovl6) | 4.19 | 10.26 | - | - | 0.36 | - |
| [AF035156](http://www.ncbi.nlm.nih.gov/entrez/viewer.fcgi?val=AF035156&view=gb) | Hydroxysteroid (17-beta) dehydrogenase 3 | [Hsd17b3](http://genecards.curie.fr/cgi-genecards/cardsearch.pl?search=Hsd17b3) | 3.82 | 0.40 | 5.84 | 2.45 | 0.84 | 0.80 |
| [AW921253](http://www.ncbi.nlm.nih.gov/entrez/viewer.fcgi?val=AW921253&view=gb) | Poly (A) polymerase alpha |  | 3.61 | 0.77 | 0.99 | 1.73 | - | 2.43 |
| [NM_019249](http://www.ncbi.nlm.nih.gov/entrez/viewer.fcgi?val=NM_019249&view=gb) | Protein tyrosine phosphatase, receptor type, F | [Ptprf](http://genecards.curie.fr/cgi-genecards/cardsearch.pl?search=Ptprf) | 3.57 | 4.78 | 1.58 | 2.74 | 2.57 | - |
| [AF292116](http://www.ncbi.nlm.nih.gov/entrez/viewer.fcgi?val=AF292116&view=gb) | Transmembrane domain protein regulated in adipocytes | [Tpra40](http://genecards.curie.fr/cgi-genecards/cardsearch.pl?search=Tpra40) | 3.54 | 1.56 | 2.92 | 3.91 | 0.49 | 0.34 |
| [AF016180](http://www.ncbi.nlm.nih.gov/entrez/viewer.fcgi?val=AF016180&view=gb) | Putative pheromone receptor (Go-VN3) | [LOC286983](http://genecards.curie.fr/cgi-genecards/cardsearch.pl?search=LOC286983) | 3.46 | 1.70 | 3.80 | 6.51 | 1.88 | 0.07 |
| [M80601](http://www.ncbi.nlm.nih.gov/entrez/viewer.fcgi?val=M80601&view=gb) | Programmed cell death 2 | [Pdcd2](http://genecards.curie.fr/cgi-genecards/cardsearch.pl?search=Pdcd2) | 3.35 | 1.31 | 5.91 | 1.21 | 8.56 | 8.92 |
| [X84004](http://www.ncbi.nlm.nih.gov/entrez/viewer.fcgi?val=X84004&view=gb) | Dual specificity phosphatase 1 | [Dusp1](http://genecards.curie.fr/cgi-genecards/cardsearch.pl?search=Dusp1) | 3.34 | 3.16 | 1.62 | 2.14 | 1.07 | 0.35 |
| [AI176627](http://www.ncbi.nlm.nih.gov/entrez/viewer.fcgi?val=AI176627&view=gb) | YTH domain family 2 |  | 3.32 | 2.23 | 21.04 | - | 1.25 | - |
| [NM_012935](http://www.ncbi.nlm.nih.gov/entrez/viewer.fcgi?val=NM_012935&view=gb) | Crystallin, alpha B | [Cryab](http://genecards.curie.fr/cgi-genecards/cardsearch.pl?search=Cryab) | 3.24 | 2.53 | 4.96 | 0.61 | 7.34 | - |

***Table 2-***continued

| **Accession**  **No.** | **Gene name** | **Gene symbol** | **E2** | **DES** | **OP** | **NP** | **BPA** | **Gen** |
| --- | --- | --- | --- | --- | --- | --- | --- | --- |
| [AF154245](http://www.ncbi.nlm.nih.gov/entrez/viewer.fcgi?val=AF154245&view=gb) | chemotactic protein-3 gene |  | 3.20 | 4.47 | 2.63 | 4.05 | 1.94 | 0.94 |
| [U55849](http://www.ncbi.nlm.nih.gov/entrez/viewer.fcgi?val=U55849&view=gb) | Tumor necrosis factor receptor superfamily, member 1b | [Tnfrsf1b](http://genecards.curie.fr/cgi-genecards/cardsearch.pl?search=Tnfrsf1b) | 3.16 | 2.49 | 0.83 | - | 0.46 | 4.21 |
| [AF200359](http://www.ncbi.nlm.nih.gov/entrez/viewer.fcgi?val=AF200359&view=gb) | UDP-glucose ceramide glucosyltransferase-like 1 | [Ugcgl1](http://genecards.curie.fr/cgi-genecards/cardsearch.pl?search=Ugcgl1) | 3.11 | 3.25 | 1.58 | 0.59 | 1.27 | 1.91 |
| [X82669](http://www.ncbi.nlm.nih.gov/entrez/viewer.fcgi?val=X82669&view=gb) | RT1 class Ib, locus Aw2 | [RT1-Aw2](http://genecards.curie.fr/cgi-genecards/cardsearch.pl?search=RT1-Aw2) | 3.03 | 3.63 | 5.47 | - | 0.43 | - |
| [NM_145788](http://www.ncbi.nlm.nih.gov/entrez/viewer.fcgi?val=NM_145788&view=gb) | TRAF family member-associated Nf-kappa B activator | [Tank](http://genecards.curie.fr/cgi-genecards/cardsearch.pl?search=Tank) | 3.00 | 3.05 | 0.95 | 1.03 | 2.45 | - |
| [Y15054](http://www.ncbi.nlm.nih.gov/entrez/viewer.fcgi?val=Y15054&view=gb) | Coronin 7 | [Loc192276](http://genecards.curie.fr/cgi-genecards/cardsearch.pl?search=Loc192276) | 2.97 | 5.16 | 3.51 | 0.82 | 1.99 | 0.93 |
| [J05571](http://www.ncbi.nlm.nih.gov/entrez/viewer.fcgi?val=J05571&view=gb) | Methionine adenosyltransferase II, alpha | [Mat2a](http://genecards.curie.fr/cgi-genecards/cardsearch.pl?search=Mat2a) | 2.92 | 5.29 | 1.39 | - | 0.93 | - |
| [X62528](http://www.ncbi.nlm.nih.gov/entrez/viewer.fcgi?val=X62528&view=gb) | Ribonuclease/angiogenin inhibitor 1 | [Rnh1](http://genecards.curie.fr/cgi-genecards/cardsearch.pl?search=Rnh1) | 2.89 | 1.21 | 1.53 | 1.76 | 2.84 | 3.95 |
| [NM_031718](http://www.ncbi.nlm.nih.gov/entrez/viewer.fcgi?val=NM_031718&view=gb) | RAB2, member RAS 3ignaling family | [Rab2](http://genecards.curie.fr/cgi-genecards/cardsearch.pl?search=Rab2) | 2.83 | 1.37 | 2.19 | - | 2.78 | 1.37 |
| [NM_022942](http://www.ncbi.nlm.nih.gov/entrez/viewer.fcgi?val=NM_022942&view=gb) | Butyrylcholinesterase | [Bche](http://genecards.curie.fr/cgi-genecards/cardsearch.pl?search=Bche) | 2.79 | 1.43 | 4.60 | 1.86 | 2.53 | 4.92 |
| [AF157005](http://www.ncbi.nlm.nih.gov/entrez/viewer.fcgi?val=AF157005&view=gb) | Myosin, heavy polypeptide 8, skeletal muscle, perinatal | [Myh8](http://genecards.curie.fr/cgi-genecards/cardsearch.pl?search=Myh8) | 2.76 | 3.94 | 1.50 | - | 1.24 | - |
| [AI385307](http://www.ncbi.nlm.nih.gov/entrez/viewer.fcgi?val=AI385307&view=gb) | Similar to tripartite motif protein 11 |  | 2.74 | 1.45 | 0.97 | 5.27 | 2.33 | 2.77 |
| [AY027880](http://www.ncbi.nlm.nih.gov/entrez/viewer.fcgi?val=AY027880&view=gb) | Stromal antigen 3 | [Stag3](http://genecards.curie.fr/cgi-genecards/cardsearch.pl?search=Stag3) | 2.72 | 0.82 | 17.04 | 1.25 | 1.46 | 2.05 |
| [NM_022849](http://www.ncbi.nlm.nih.gov/entrez/viewer.fcgi?val=NM_022849&view=gb) | deleted in malignant brain tumors 1 | Dmbt1 | 2.70 | 1.30 | 2.43 | 2.84 | 2.53 | 0.97 |
| [AJ278701](http://www.ncbi.nlm.nih.gov/entrez/viewer.fcgi?val=AJ278701&view=gb) | Branched chain aminotransferase 1, cytosolic | [Bcat1](http://genecards.curie.fr/cgi-genecards/cardsearch.pl?search=Bcat1) | 2.68 | 3.30 | 3.42 | - | 4.78 | 0.25 |
| [NM_017341](http://www.ncbi.nlm.nih.gov/entrez/viewer.fcgi?val=NM_017341&view=gb) | Lipase, gastric | [Lipf](http://genecards.curie.fr/cgi-genecards/cardsearch.pl?search=Lipf) | 2.67 | 1.86 | 2.44 | 7.62 | 1.84 | - |
| [AB081072](http://www.ncbi.nlm.nih.gov/entrez/viewer.fcgi?val=AB081072&view=gb) | COP9 (constitutive photomorphogenic) homolog, subunit 2 | [Cops2](http://genecards.curie.fr/cgi-genecards/cardsearch.pl?search=Cops2) | 2.66 | 1.59 | 0.82 | - | 1.94 | 2.23 |
| [BF563877](http://www.ncbi.nlm.nih.gov/entrez/viewer.fcgi?val=BF563877&view=gb) | Similar to erythroid differentiation-related factor 1 |  | 2.66 | - | 1.94 | 2.58 | 1.63 | - |
| [X89999](http://www.ncbi.nlm.nih.gov/entrez/viewer.fcgi?val=X89999&view=gb) | Sperm adhesion molecule | [Spam](http://genecards.curie.fr/cgi-genecards/cardsearch.pl?search=Spam) | 2.65 | 0.64 | 1.26 | - | 0.79 | - |
| [NM_053713](http://www.ncbi.nlm.nih.gov/entrez/viewer.fcgi?val=NM_053713&view=gb) | Kruppel-like factor 4 (gut) | [Klf4](http://genecards.curie.fr/cgi-genecards/cardsearch.pl?search=Klf4) | 2.64 | 5.24 | 2.82 | 1.89 | 1.96 | - |
| [NM_031029](http://www.ncbi.nlm.nih.gov/entrez/viewer.fcgi?val=NM_031029&view=gb) | Gamma-aminobutyric acid (GABA) A receptor, pi | [Gabrp](http://genecards.curie.fr/cgi-genecards/cardsearch.pl?search=Gabrp) | 2.59 | 2.90 | 0.59 | - | 1.19 | 0.41 |
| [NM_017007](http://www.ncbi.nlm.nih.gov/entrez/viewer.fcgi?val=NM_017007&view=gb) | Glutamate decarboxylase 1 | [Gad1](http://genecards.curie.fr/cgi-genecards/cardsearch.pl?search=Gad1) | 2.58 | 3.51 | 5.28 | - | 1.62 | 1.75 |
| [AF022085](http://www.ncbi.nlm.nih.gov/entrez/viewer.fcgi?val=AF022085&view=gb) | Similar to guanine nucleotide binding protein beta 4 |  | 2.58 | 1.11 | 2.15 | 2.83 | 1.25 | 0.25 |
| [NM_031750](http://www.ncbi.nlm.nih.gov/entrez/viewer.fcgi?val=NM_031750&view=gb) | Heat shock 27kD protein family, member 3 | [Hspb3](http://genecards.curie.fr/cgi-genecards/cardsearch.pl?search=Hspb3) | 2.56 | 1.03 | 1.07 | 0.76 | - | 3.13 |
| [X57523](http://www.ncbi.nlm.nih.gov/entrez/viewer.fcgi?val=X57523&view=gb) | Transporter 1, ATP-binding cassette, sub-family B (MDR/TAP) | [Tap1](http://genecards.curie.fr/cgi-genecards/cardsearch.pl?search=Tap1) | 2.51 | 1.72 | 1.16 | 1.38 | 4.09 | 3.08 |
| [BF414914](http://www.ncbi.nlm.nih.gov/entrez/viewer.fcgi?val=BF414914&view=gb) | Similar to Mouse primary response gene B94 mRNA, 3end. |  | 2.49 | 6.65 | 0.35 | - | 1.20 | 2.18 |
| [AF272892](http://www.ncbi.nlm.nih.gov/entrez/viewer.fcgi?val=AF272892&view=gb) | Corneal wound healing related protein | [Mak10](http://genecards.curie.fr/cgi-genecards/cardsearch.pl?search=Mak10) | 2.46 | 1.89 | 1.12 | 5.65 | 2.76 | 1.03 |
| [NM_019157](http://www.ncbi.nlm.nih.gov/entrez/viewer.fcgi?val=NM_019157&view=gb) | Aquaporin 7 | [Aqp7](http://genecards.curie.fr/cgi-genecards/cardsearch.pl?search=Aqp7) | 2.45 | 0.21 | 4.10 | 4.28 | 0.62 | 0.51 |
| [NM_013049](http://www.ncbi.nlm.nih.gov/entrez/viewer.fcgi?val=NM_013049&view=gb) | Tumor necrosis factor receptor superfamily, member 4 | [Tnfrsf4](http://genecards.curie.fr/cgi-genecards/cardsearch.pl?search=Tnfrsf4) | 2.45 | 2.92 | 2.26 | 0.44 | 1.40 | 2.65 |
| [NM_031056](http://www.ncbi.nlm.nih.gov/entrez/viewer.fcgi?val=NM_031056&view=gb) | Matrix metalloproteinase 14 (membrane-inserted) | [Mmp14](http://genecards.curie.fr/cgi-genecards/cardsearch.pl?search=Mmp14) | 2.40 | 1.90 | 1.58 | 1.92 | 4.96 | 4.72 |
| [NM_031693](http://www.ncbi.nlm.nih.gov/entrez/viewer.fcgi?val=NM_031693&view=gb) | synaptotagmin 4 | Syt4 | 2.40 | 0.97 | 2.43 | 2.74 | 0.82 | - |
| [AF140232](http://www.ncbi.nlm.nih.gov/entrez/viewer.fcgi?val=AF140232&view=gb) | S100 calcium binding protein A6 (calcyclin) | [S100a6](http://genecards.curie.fr/cgi-genecards/cardsearch.pl?search=S100a6) | 2.39 | 3.32 | 3.52 | 2.31 | 0.82 | 0.64 |
| [Y13972](http://www.ncbi.nlm.nih.gov/entrez/viewer.fcgi?val=Y13972&view=gb) | MHC class I-like sequence | [Hlals](http://genecards.curie.fr/cgi-genecards/cardsearch.pl?search=Hlals) | 2.38 | 2.47 | 1.65 | - | 3.62 | 1.00 |
| [AJ245707](http://www.ncbi.nlm.nih.gov/entrez/viewer.fcgi?val=AJ245707&view=gb) | 2-hydroxyphytanoyl-Coenzyme A lyase | [Hpcl2](http://genecards.curie.fr/cgi-genecards/cardsearch.pl?search=Hpcl2) | 2.38 | 1.37 | 2.82 | 1.52 | 2.60 | 2.00 |
| [AB030238](http://www.ncbi.nlm.nih.gov/entrez/viewer.fcgi?val=AB030238&view=gb) | hepatocarcinogenesis-related transcription factor | HTF | 2.38 | 3.60 | 3.28 | 0.72 | 1.07 | 1.30 |
| [NM_024402](http://www.ncbi.nlm.nih.gov/entrez/viewer.fcgi?val=NM_024402&view=gb) | A-kinase anchor protein 4 | [Akap4](http://genecards.curie.fr/cgi-genecards/cardsearch.pl?search=Akap4) | 2.37 | 1.89 | - | - | 2.67 | 4.04 |
| [L26288](http://www.ncbi.nlm.nih.gov/entrez/viewer.fcgi?val=L26288&view=gb) | Regulator of G-protein 3ignaling 19 | [Camk1](http://genecards.curie.fr/cgi-genecards/cardsearch.pl?search=Camk1) | 2.37 | 2.77 | 4.90 | 1.08 | 2.20 | 1.72 |
| [AF239157](http://www.ncbi.nlm.nih.gov/entrez/viewer.fcgi?val=AF239157&view=gb) | RAS, dexamethasone-induced 1 | [Rasd1](http://genecards.curie.fr/cgi-genecards/cardsearch.pl?search=Rasd1) | 2.36 | 4.69 | 4.42 | 1.59 | 5.38 | 0.81 |
| [U55816](http://www.ncbi.nlm.nih.gov/entrez/viewer.fcgi?val=U55816&view=gb) | Solute carrier family 12,  (potassium-chloride transporter) member 5 | [Slc12a5](http://genecards.curie.fr/cgi-genecards/cardsearch.pl?search=Slc12a5) | 2.32 | - | 1.17 | 1.99 | 0.83 | 5.66 |
| [NM_013057](http://www.ncbi.nlm.nih.gov/entrez/viewer.fcgi?val=NM_013057&view=gb) | Coagulation factor 3 | [F3](http://genecards.curie.fr/cgi-genecards/cardsearch.pl?search=F3) | 2.31 | 0.90 | 1.54 | 3.11 | 2.25 | - |
| [NM_017025](http://www.ncbi.nlm.nih.gov/entrez/viewer.fcgi?val=NM_017025&view=gb) | Lactate dehydrogenase A | [Ldha](http://genecards.curie.fr/cgi-genecards/cardsearch.pl?search=Ldha) | 2.17 | 2.06 | 1.77 | 2.72 | 0.98 | 0.98 |
| [NM_031698](http://www.ncbi.nlm.nih.gov/entrez/viewer.fcgi?val=NM_031698&view=gb) | Ribophorin 2 |  | 2.17 | 1.12 | 1.27 | 2.21 | 1.67 | 0.75 |
| [AF151982](http://www.ncbi.nlm.nih.gov/entrez/viewer.fcgi?val=AF151982&view=gb) | Secretory leukocyte peptidase inhibitor | [Slpi](http://genecards.curie.fr/cgi-genecards/cardsearch.pl?search=Slpi) | 2.11 | 2.89 | 2.39 | 2.25 | 0.98 | 0.94 |
